# Supplementary material for: Combined Effects of Surface Roughness, Solubility Parameters, and Hydrophilicity on Biofouling of Reverse Osmosis Membranes
Source: Membranes (Basel). 2024 Nov 8;14(11):235. doi: 10.3390/membranes14110235 (PMC11596770; doi:10.3390/membranes14110235)
Supplement: Supplementary file 1 [file membranes-14-00235-s001.zip › membranes-3225285-supplementary.pdf]

membranes

MDPI

Supplementary Materials

Combined Effects of Surface Roughness, Solubility Parameters, and Hydrophilicity on Biofouling of Reverse Osmosis Membranes

Neveen AlQasas and Daniel Johnson *

Water Research Center (WRC), Division of Engineering, New York University Abu Dhabi, Abu Dhabi P.O. Box 129188, United Arab Emirates; na3150@nyu.edu
* Correspondence: djj2026@nyu.edu

Table S1. The specification of the commercial membranes used in the study.

No.	Commercial Name	Feed type	Flux (gfd/psi)	Salt rejection	Active layer material	Manufacturer	pH range (25C)
1	UTC-73HA-PA-RO	Brackish water	23.3/73	99% NaCl	Polyamide-TFC	Toray	2–11
2	X201, PA-UREA, RO	Industrial/Wastewater	30/225	99.5%	Polyamide-urea-TFC	TriSepTM	2–11
3	SW30XLE-PA-TFC-RO	Seawater RO	18.1/800	99.75% NaCl	Polyamide-TFC	FilmTec™	1–13
4	XLE-PA-TFC-RO	Brackish water	22.4/225	98.7% NaCl	Polyamide-TFC	FilmTec™	2–11
5	AK-TFC RO	Brackish Water	26/115	99.0% NaCl	Polyamide-TFC	Suez (GE)™	1–11
6	BW30XFRLE	Industrial/Wastewater	28.8/150	99.3%	Polyamide-TFC	FilmTec™	1–13
7	BW30-PA-TFC-RO	Brackish Water	30/220	99.7%	Polyamide-TFC	FilmTec™	2–11
8	CR100	Chemical/Petrochemical	28.8/225	99.7% NaCl	Polyamide-TFC	FilmTec™	1–13

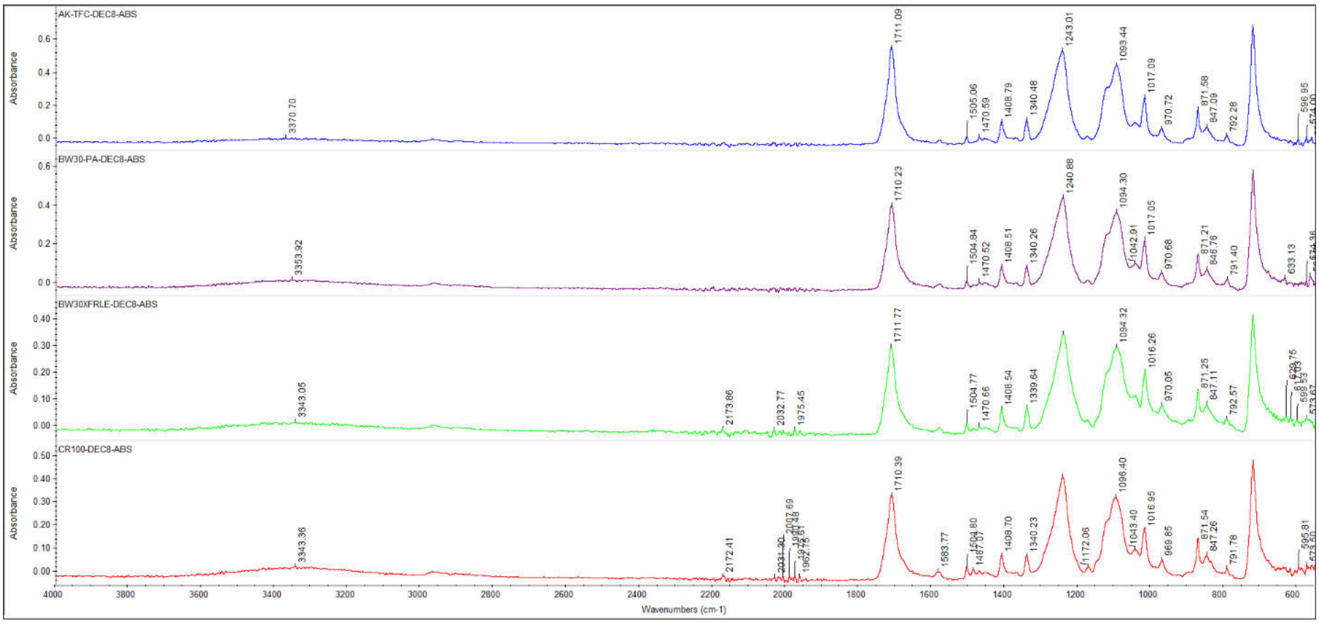

Figure S1. The FTIR results for four commercial membranes, AK-TFC, Bw30-PA, BW30FRLE, and CR100.

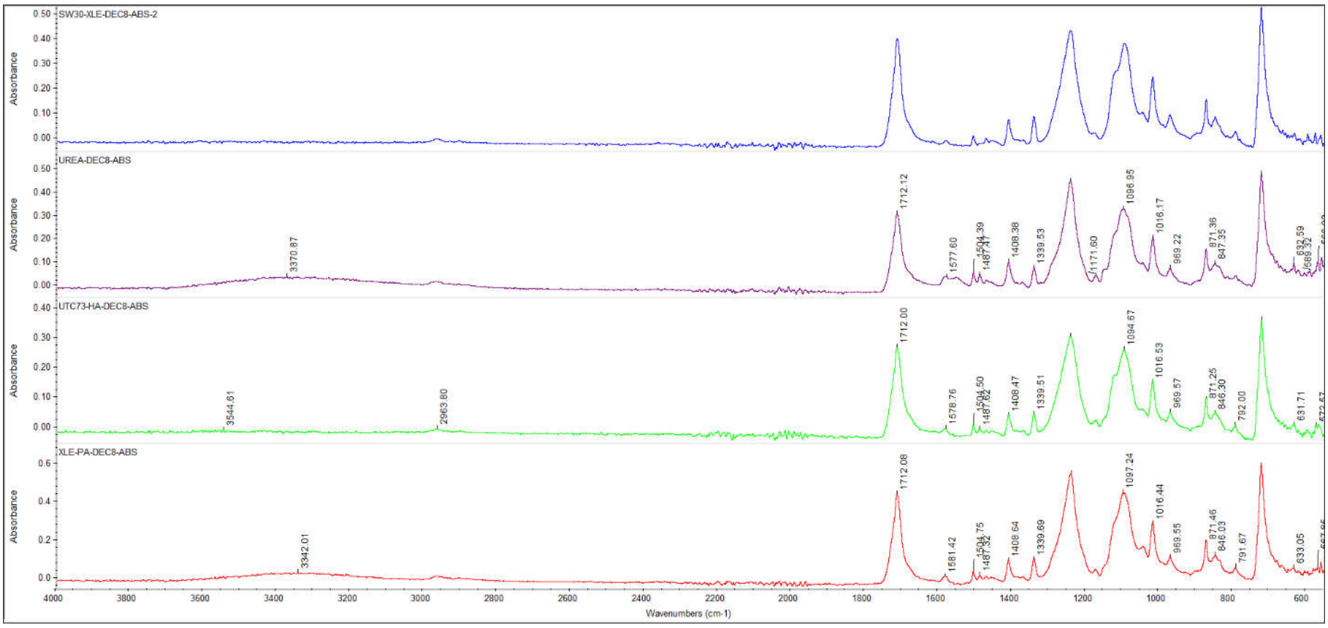

Figure S2. The FTIR results for four commercial membranes, SW30-XLE, UREA, UTC73-HA, and XLE-PA.

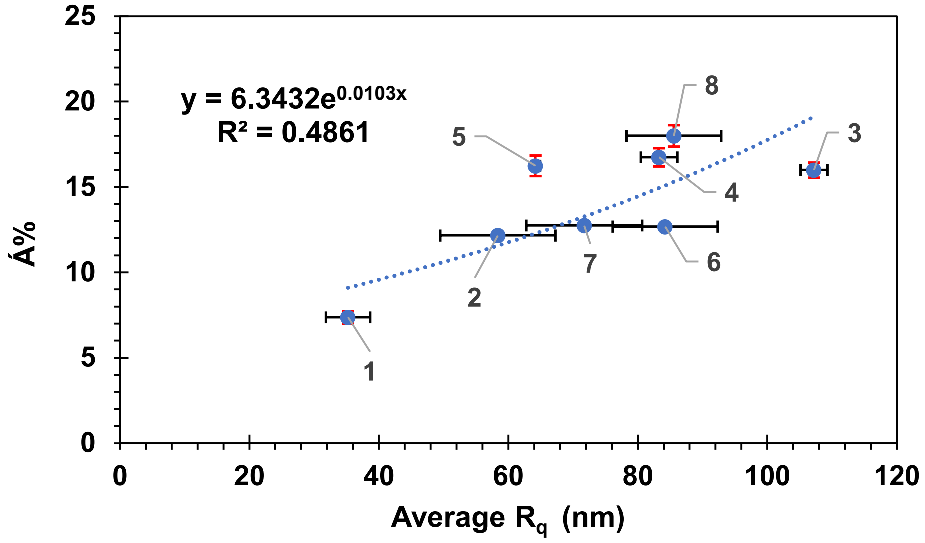

Figure S3. The surface area percentage difference (Á %) versus R_q. Error bars show the standard error. Each membrane is numbered according to the scheme in Table 2.

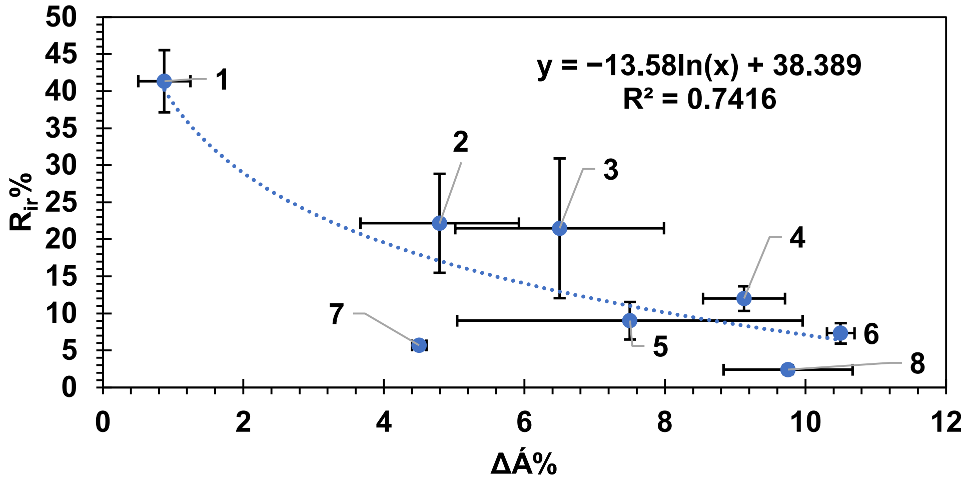

Figure S4. The change in surface area percentage difference between fouled and pristine membrane versus the flux recovery ratio on each of the membranes. Each membrane is numbered according to the scheme in Table 2. Error bars show the standard error.

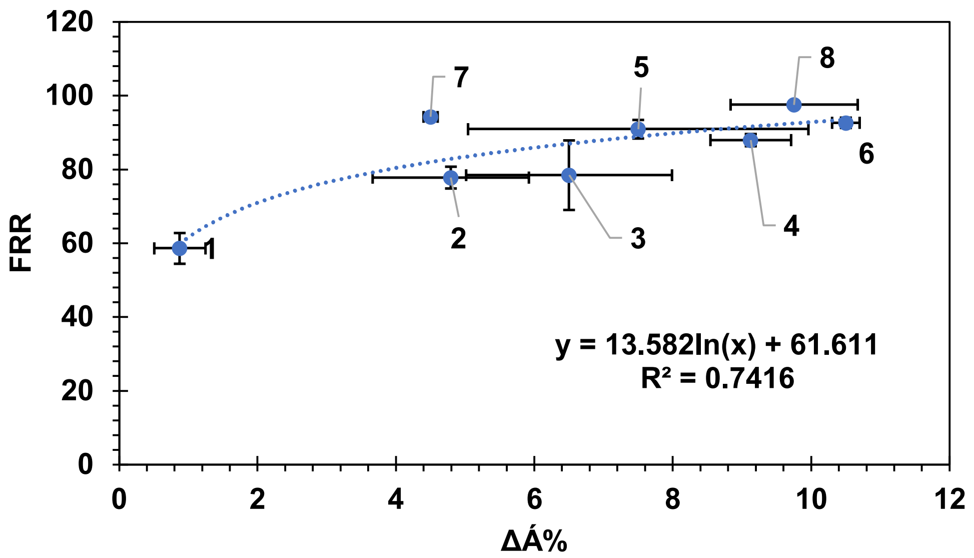

Figure S5. The change in surface area percentage difference between fouled and pristine membrane versus the percentage of irreversible fouling on each of membranes. Each membrane is numbered according to the scheme in Table 2. Error bars show the standard error.

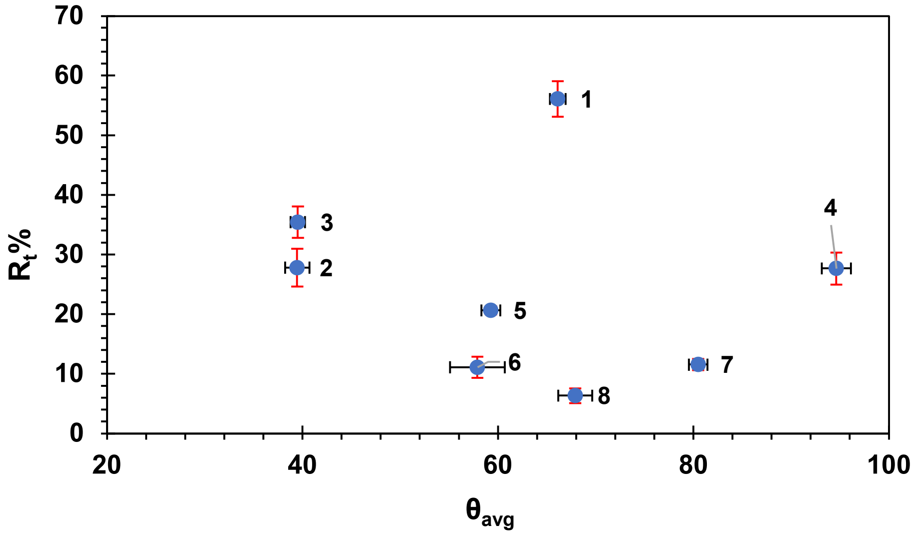

Figure S6. The Total fouling percentage R_T% versus the average water contact angle (θ_{avg}) on the membranes. Each membrane is numbered according to the scheme in Table 2. Error bars show the standard error.
